# Automated Speech Analysis for Screening and Monitoring Bipolar Depression: Machine Learning Model Development and Interpretation Study

**DOI:** 10.2196/79093

**Published:** 2025-12-04

**Authors:** Sooyeon Min, Tae-Sung Yeum, Daun Shin, Sang Jin Rhee, Hyunju Lee, Han-Sung Lee, Seongmin Park, Jihwa Lee, Yong Min Ahn

**Affiliations:** 1 Department of Neuropsychiatry Seoul National University Hospital Seoul Republic of Korea; 2 Department of Psychiatry and Behavioral Science College of Medicine Seoul National University Seoul Republic of Korea; 3 Department of Psychiatry Korea University Medical Center Seoul Republic of Korea; 4 ActionPower Corp. Seoul Republic of Korea

**Keywords:** depression, voice analysis, speech modalities, bipolar disorder, artificial intelligence, AI, natural language processing

## Abstract

**Background:**

Depressive episodes in bipolar disorder are frequent, prolonged, and contribute substantially to functional impairment and reduced quality of life. Therefore, early and objective detection of bipolar depression is critical for timely intervention and improved outcomes. Multimodal speech analyses hold promise for capturing psychomotor, cognitive, and affective changes associated with bipolar depression.

**Objective:**

This study aims to develop between- and within-person classifiers to screen for bipolar depression and monitor longitudinal changes to detect depressive recurrence in patients with bipolar disorder. A secondary objective was to compare the predictive performance across speech modalities.

**Methods:**

We collected 304 voice audio recordings obtained during semistructured interviews with 92 patients diagnosed with bipolar disorder over a 1-year period. Depression severity was assessed using the Hamilton Depression Rating Scale. Acoustic features were extracted using the openSMILE toolkit, and linguistic features were extracted using the Linguistic Inquiry and Word Count frameworks following automatic speech recognition and machine translation. Mixed-effects multivariate linear regression evaluated the associations between speech markers and Hamilton Depression Rating Scale scores adjusting for demographic variables, diagnosis, and feature-specific covariates. Extreme gradient boosting and light gradient boosting were used as base learners. We developed a between-person classifier to detect moderate to severe depression and a within-person classifier to detect recurrence. Hyperparameter tuning and 95% CI estimation were performed using a bootstrap bias-corrected cross-validation (*k*=5) approach combined with a grid search. Feature contributions were interpreted using Shapley additive explanations.

**Results:**

Patients with depression showed reduced energy modulation, prolonged monotony, and more frequent use of words related to death and negative emotions. The between-person classifier combining acoustic and linguistic features detected moderate to severe depression with an area under the curve of 0.76 compared to 0.54 for the demographic model. The within-person classifier based on speech features detected depression recurrence with an area under the curve of 0.70 compared to 0.55 for the demographic model.

**Conclusions:**

Between- and within-person comparisons of speech markers can be leveraged in detecting and monitoring bipolar depression. We demonstrate the feasibility of applying Linguistic Inquiry and Word Count–based psycholinguistic analysis to machine–transcribed and translated speech, supporting the replicability of this approach across languages. Automated multimodal voice analysis can be integrated into digital health platforms, providing a scalable and effective approach for accessing mental health monitoring and care.

## Introduction

Depression affects more than 280 million individuals worldwide and is a leading contributor to the global burden of disease [1,2]. As diagnosis relies entirely on symptomatology without the support of laboratory tests or imaging, clinicians must depend on subjective evaluations of patients’ reported experiences and observable behaviors [3], which poses challenges for accurate and timely diagnosis.

Clinicians have long observed speech changes in depression, such as reduced pitch variation, slower speech rate, and diminished output [4,5]. Speech has acoustic features (eg, lower pitch and narrower pitch range) [5,6], which may reflect psychomotor or autonomic alterations, and linguistic features (eg, increased use of first-person singular pronouns and negative-sentiment words) [7], which may reflect cognitive and affective disturbances. Voice data offer several advantages: they are noninvasive, low cost, and suitable for repeated assessments even in remote or resource-limited settings. Furthermore, voice is also sensitive to within-person mood fluctuations, making it well suited for longitudinal monitoring [8]. Recent advances have increased sophistication in voice-based depression detection, with growing adoption of multimodal approaches [9-11] and application of deep learning–based models [12].

A recent systematic review highlighted the promising potential of voice markers in the detection of depression [13], reporting classification performance with an area under the receiver operating characteristic curve (AUC) ranging from 0.71 to 0.93. However, substantial methodological heterogeneity was identified across the studies, including patient selection and diagnosis based on clinician- or self-rated symptom scales. Many studies have not validated models using cross-validation or an independent test set. Some studies have explored changes in speech patterns associated with changes in depression severity [14] or clinical outcomes after treatment [15-17], but they are limited in modality—primarily acoustic—and duration, typically involving 4 to 6 weeks of follow-up. To the best of our knowledge, no previous study has systematically compared the cross-sectional and longitudinal performance of acoustic and linguistic speech markers in a clinically diagnosed sample and assessed their utility for ongoing symptom monitoring in real-world practice.

While much of this research has focused on major depressive disorder (MDD), depressive episodes are also the predominant and most debilitating feature of bipolar disorder. Although bipolar disorder is defined by the presence of manic or hypomanic episodes, longitudinal studies have consistently shown that patients spend most of their illness course in depressive states rather than manic or hypomanic phases [18-20]. These depressive episodes are frequent and prolonged and contribute disproportionately to functional impairment, suicide risk, and reduced quality of life. Accordingly, developing objective methods for detecting and monitoring bipolar depression is of high clinical importance.

In this prospective study, we collected voice recordings from 92 individuals with a psychiatrist-confirmed diagnosis of bipolar disorder over a 1-year period. The main objective of this study was to develop machine learning–based classification models for detecting and monitoring bipolar depression using voice recordings. A between-person classifier that compared the severity of depression between individual recordings and a within-person classifier that compared changes in severity within the same individual were developed. The secondary objective was to compare the acoustic, linguistic, and combined speech features to detect and monitor depressive symptoms.

## Methods

### Study Participants and Clinical Assessments

Adult patients (aged ≥19 years) diagnosed with bipolar disorder (type 1, type 2, and not otherwise specified) attending the Mood and Anxiety Clinic at Seoul National University Hospital were recruited through convenience sampling. Board-certified psychiatrists confirmed the psychiatric diagnoses. The exclusion criteria included major psychiatric disorders other than bipolar disorder, laryngeal conditions or surgeries that could impair voice production, and psychiatric symptoms attributable to organic brain diseases or previous intracranial surgery.

Clinical evaluations were conducted at baseline and repeated at 2, 4, 8, and 12 months. Demographic and clinical data, including age, sex, height, weight, comorbidities, and prescribed medications, were collected at baseline, and changes were tracked at each follow-up visit. Cumulative antipsychotic dosages were standardized to the equivalent dose of aripiprazole [21,22], which was the predominant antipsychotic prescribed in the study sample. Depressive symptom severity was assessed using the Korean adaptation of the 17-item Hamilton Depression Rating Scale (HAM-D) [23,24], administered by trained raters. Each HAM-D item is scored from 0 (not present) to 4 (severe), yielding total scores ranging from 0 to 68. Standard clinical cutoffs classify scores of ≤7 as minimal depression, scores of 8 to 16 as mild depression, scores of 17 to 23 as moderate depression, and scores of ≥24 as severe depression [25]. The patients also self-rated suicidality, depression, and anxiety symptoms using the Korean version of the Beck Scale for Suicide Ideation, Patient Health Questionnaire-9 items, and Beck Anxiety Inventory. A more detailed study protocol has been published previously [22].

### Preprocessing of Voice Data and Speech Feature Extraction

Study participants’ voices were recorded during semistructured psychiatric interviews conducted by 1 of the 2 research nurses using a Sony ICD-SX813 voice recorder in a standardized interview room setting [22]. While interview structure ensured consistency across participants, interviewers were encouraged to use open-ended prompts. Segments with the interviewer’s voice, overlapping speech or background noise, and utterances shorter than 3 seconds were excluded. Audio files were transcribed using the Daglo application programming interface (ActionPower Corp), a conformer-based automatic speech recognition engine [26-28] recognized for its superior accuracy in Korean speech recognition tasks. We manually reviewed 2% of the automatically generated transcripts and compared them to their corresponding audio recordings to verify transcription accuracy and calculate the character error rate (CER). CER quantifies transcription errors as the percentage of incorrectly transcribed characters (including substitutions, deletions, and insertions) normalized by the total character count [29].

The acoustic features were extracted using the INTERSPEECH 2016 Computational Paralinguistics Challenge (ComParE 2016) feature set, the largest available set in the openSMILE toolkit (audEERING GmbH) [30-32], which is widely used for depression detection because of its well-documented and standardized feature extraction process [31,33-35]. A total of 6373 features were extracted per recording, covering functionals derived from low-level descriptors such as pitch (F0), loudness, mel-frequency cepstral coefficients, jitter, shimmer, and spectral features.

Linguistic features were extracted using a Linguistic Inquiry and Word Count (LIWC) framework (Pennebaker Conglomerates, Inc). The LIWC framework calculates the frequency of words within psychologically relevant categories serving as proxies for various social, cognitive, and affective processes. It is one of the most widely used psycholinguistic dictionaries in natural language processing tasks [36,37]. For this analysis, we used the LIWC and the LIWC Extension framework (Receptiviti) [38]. As the Korean LIWC library was unavailable at the time of our study, we machine translated texts using the DeepL application programming interface (DeepL SE) from Korean to English and then assigned words to categories using the LIWC frameworks in English [39,40]. LIWC scores range between 0 and 1 and denote the proportion of words in a specific category relative to the total word count in the text.

### Statistical Analyses and Development of Machine Learning Classifiers

Given the repeated-measurement structure, we applied mixed-effects multivariate linear regression models to identify the speech features associated with the HAM-D score after applying an inverse normal transformation. Features that were constant across all recordings (such as the “parentheses” LIWC category) were excluded from analysis. Covariates included age, sex, BMI, diagnosis, and antipsychotic dosage for acoustic feature analysis and age, sex, years of education, and diagnosis for linguistic analysis. The significance level was set at *P*<.05, and the Benjamini-Hochberg test was conducted to correct for multiple hypotheses. All statistical analyses were conducted using R (version 4.1.0; R Foundation for Statistical Computing).

All acoustic and linguistic features were used for classifier development. Decision tree classifiers were selected for their robustness and established performance in previous studies [22,41,42]. We used extreme gradient boosting (XGB) and light gradient boosting (LGB) as the base learner algorithms. XGB and LGB are both gradient boosting frameworks. LGB is noted for its computational efficiency, whereas XGB is renowned for its fast convergence speed.

To address class imbalance at both the data and algorithmic levels, we combined the synthetic minority oversampling technique (SMOTE) with the focal loss function. SMOTE was applied exclusively to the training set within each cross-validation fold to generate synthetic samples of the minority class while preventing data leakage. In parallel, focal loss was implemented in both the LGB and XGB models to further mitigate imbalance during model optimization. The α parameter of focal loss was automatically calculated based on class ratios to balance positive and negative samples, and the γ parameter was fixed at 2.0 to emphasize harder-to-classify samples.

Hyperparameter tuning and 95% CIs were estimated using a bootstrap bias-corrected cross-validation (BBCV; *k*=5) approach. BBCV involves nonparametric bootstrapping with 1000 iterations on the out-of-sample predictions from the validation folds combined with a grid search for hyperparameter optimization [43]. BBCV provides unbiased and conservative estimates of model performance, particularly in studies with small sample sizes, and it may outperform alternatives such as nested cross-validation. The model performance was evaluated using the AUC. The accuracy, sensitivity, specificity, and positive predictive value at the optimal threshold defined by the Youden index (sensitivity + specificity − 1) were also reported.

The participants were categorized as having moderate to severe depression if their HAM-D score was ≥17 [25]. The HAM-D is the gold-standard instrument for evaluating antidepressant treatment efficacy, with a ≥50% reduction in score considered a conventional criterion for treatment response [44]. Although there is no established consensus regarding the operational definition of relapse or recurrence in depression, we defined recurrence as a transition from minimal or mild depression (HAM-D<17) to moderate or severe depression (HAM-D≥17) between consecutive visits. In this paper, we use the term *recurrence* to refer to such worsening without formally distinguishing it from *recurrence* as defined in the *Diagnostic and Statistical Manual of Mental Disorders, Fifth Edition* [3]. The following two models were developed: (1) a between-person model to classify patients with moderate to severe depression and (2) a within-person model to detect recurrence since the last visit. All recordings were treated as individual data points with the goal of cross-sectional screening for severe depression. Within-person models used changes in voice features between time intervals to predict recurrence. Five-fold GroupKFold cross-validation was used to perform cross-validation at the participant level and prevent data leakage. To evaluate multimodal integration, we tested the following five feature sets: (1) age and sex variables only (referred to as “demographic”); (2) acoustic features only (referred to as “acoustic”); (3) linguistic features only (referred to as “linguistic”); (4) acoustic and linguistic features combined (referred to as “speech”); and (5) age, sex, and speech features (referred to as “speech+demographic”). Shapley additive explanation (SHAP) analysis was conducted to interpret feature contributions for each prediction task [45].

Machine learning experiments were conducted on a computing cluster with Intel Xeon Gold 6138 central processing units, NVIDIA RTX A6000 graphics processing units (48-GB video RAM), and 128-GB RAM. Analyses used Python (version 3.9.6; Python Software Foundation) with the scikit-learn (version 1.6.1; Google Summer of Code project), *lightgbm* (version 4.6.0), and *xgboost* (version 3.0.2) packages.

### Ethical Considerations

This study was approved by the institutional review board of Seoul National University Hospital (1812-081-995) and conducted in accordance with the ethical standards of the 1964 Declaration of Helsinki and its later amendments. Written informed consent was obtained from all participants during their initial visit, which included information about the study purpose, data use, and the right to withdraw at any time. All audio files were anonymized and securely stored and are not to be used for any purpose beyond the scope of consent. No identifiable information appears in the manuscript or supplementary materials, and participants are not recognizable in any shared data or figures. Participants received approximately US $20 per visit as reimbursement for transportation and time in line with standard research practices.

All participants in our study were receiving ongoing care at our outpatient psychiatric clinic throughout the study period, ensuring continuous clinical monitoring and appropriate treatment independent of the research protocol. Given that bipolar disorder is associated with high suicide risk [46], the research nurses were instructed to immediately notify the attending psychiatrist if a participant appeared to be at high risk during the interview.

## Results

### Study Participants and Speech Recordings

Participants (N=92; 70/92, 76% female) were outpatients visiting the Mood and Anxiety Clinic at Seoul National University Hospital for mild to severe depressive symptoms. After the baseline interview, 72% (66/92) of the participants attended the 2-month follow-up, 62% (57/92) attended at 4 months, 50% (46/92) attended 8 months, and 47% (43/92) attended at 12 months. In total, we collected 304 recordings, and the study participants collectively spoke for approximately 55 hours (197,081 seconds) and said 323,280 words. The average length of the concatenated patients’ voice segments at each interview session was 648.69 (SD 486.4) seconds and included 1063 (SD 833.5) words. The average sentence was 15.91 (SD 5.9) words. When manually reviewing 2% of the randomly selected machine-generated transcripts against their corresponding audio recordings, we identified a CER below 1% (range 0.02%-1.08%). There was no significant correlation between CER and HAM-D scores (*r*=0.10; *P*=.83). In total, 6373 acoustic features and 105 linguistic features were extracted.

**Figure 1:**
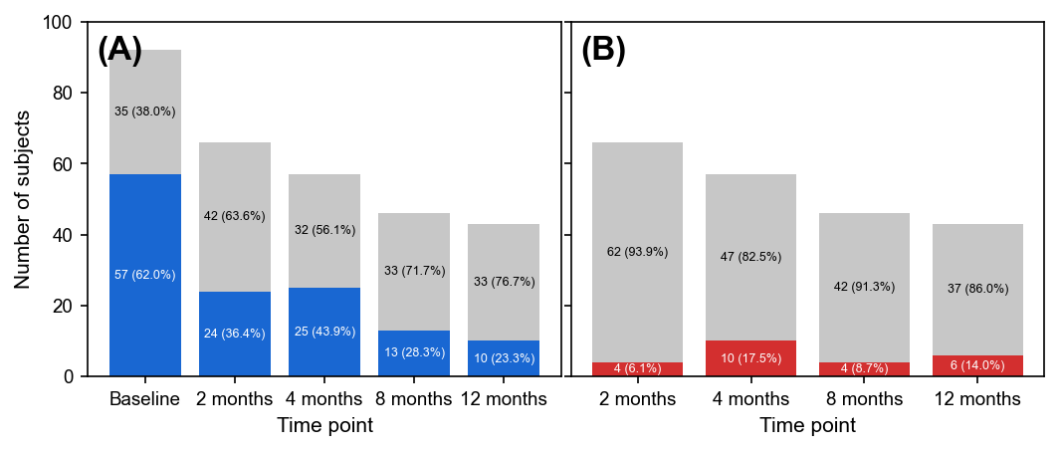
Distribution of depression severity and change in depressive symptoms over time. (A) The blue bars represent participants with moderate to severe depression, whereas the gray bars represent participants with minimal to mild depression. (B) The red bars represent participants who had recurrence, and the gray bars represent those whose symptoms remained the same or improved since their last visit.

At baseline, 62% (57/92) of the participants had moderate to severe depression. A total of 36% (24/66) at 2 months, 44% (25/57) at 4 months, 28% (13/46) at 8 months, and 23% (10/43) at 12 months were moderately to severely depressed (Figure 1A). At baseline, there were no statistically significant differences in sex (*P*=.54), BMI (*P*=.84), psychiatric diagnosis (*P*=.54), socioeconomic status (*P*=.64), years of education (*P*=.61), antipsychotic dosage (*P*=.40), and medical comorbidities (*P*=.20) between patients with bipolar disorder with moderate to severe depression and their counterparts with minimal to mild depression (Table 1). Patients with more severe depressive symptoms had greater self-reported depressive, anxiety, and suicidal symptoms (*P*<.001 in all cases). At baseline, 89% (82/92) of the participants were prescribed antipsychotics; 94% (86/92) were prescribed mood stabilizers; 30% (28/92) were prescribed antidepressants; 67% (62/92) were prescribed benzodiazepines; and 46% (42/92) were prescribed other medications, such as propranolol and benztropine. The type and dosage of medications were modified by the attending psychiatrist at each visit. Of the 92 participants, 14 (15%) completed only 1 visit, 13 (14%) completed 2 visits, 22 (24%) completed 3 visits, 17 (19%) completed 4 visits, and 26 (28%) completed all 5 visits. In 11.3% (24/212) of cases, participants had recurrence at follow-up (Figure 1B).

**Table 1:** Baseline characteristics of the groups with moderate to severe depression and minimal to mild depression.

Characteristic		Moderate to severe depression (n=57)	Minimal to mild depression (n=35)	*P* value	
Age (y), mean (SD)		29.28 (9.53)	31.97 (12.45)	.25	
**Sex, n (%)**					.54
	Male	11 (19)	10 (29)		
	Female	46 (81)	25 (71)		
BMI (kg/m^2^), mean (SD)		24.63 (4.99)	24.85 (4.95)	.84	
**Diagnosis, n (%)**					.54
	Bipolar disorder type 1	8 (14)	6 (17)		
	Bipolar disorder type 2	39 (68)	26 (74)		
	Bipolar disorder NOS^a^	10 (18)	3 (9)		
**Marital status, n (%)**					.69
	Single	43 (75)	27 (77)		
	Married	12 (21)	7 (20)		
	Divorced or widowed	2 (4)	1 (3)		
Household income (KRW), median (IQR)		2,300,000 (1,978,147-5,000,000)^b^	2,272,500 (2,036,911-5,000,000)^c^	.92	
**SES^d^, n (%)**					.64
	Very low	1 (2)	2 (6)		
	Low	10 (18)	7 (20)		
	Middle	31 (54)	15 (43)		
	High	8 (14)	4 (11)		
	Very high	7 (12)	7 (20)		
Years of education, mean (SD)		13.89 (2.14)	14.14 (2.21)	.61	
Antipsychotic dosage (mg)^e^, median (IQR)		7.50 (5-15)	5.00 (2.25-12.7)	.40	
**Medical comorbidity, n (%)**					.20
	None	43 (75)	28 (80)		
	Mild	10 (18)	3 (9)		
	Moderate	2 (4)	4 (11)		
	Severe	2 (4)	0 (0)		
History of suicide attempts, n (%)		10 (18)	3 (9)	.36	
HAM-D^f^ score (range 0-68), mean (SD)		20.63 (2.21)	12.97 (2.92)	<.001	
SSI^g^ score (range 0-38), mean (SD)		22.17 (8.01)	13.06 (8.01)	<.001	
PHQ-9^h^ score (range 0-27), mean (SD)		18.39 (5.01)	12.68 (6.17)	<.001	
BAI^i^ score (range 0-63), mean (SD)		28.89 (15.11)	17.40 (14.39)	<.001	

^a^NOS: not otherwise specified.

^b^US $1769 (US $1522-$3846).

^c^US $1748 (US $1567-$3846).

^d^SES: socioeconomic status; based on the Hollingshead-Redlich index.

^e^Antipsychotic dosage converted into the equivalent dose of aripiprazole.

^f^HAM-D: Hamilton Depression Rating Scale.

^g^SSI: Beck Scale for Suicide Ideation.

^h^PHQ-9: Patient Health Questionnaire-9 items.

^i^BAI: Beck Anxiety Inventory.

### Acoustic and Linguistic Features Associated With Depression Severity

**Figure 2:**
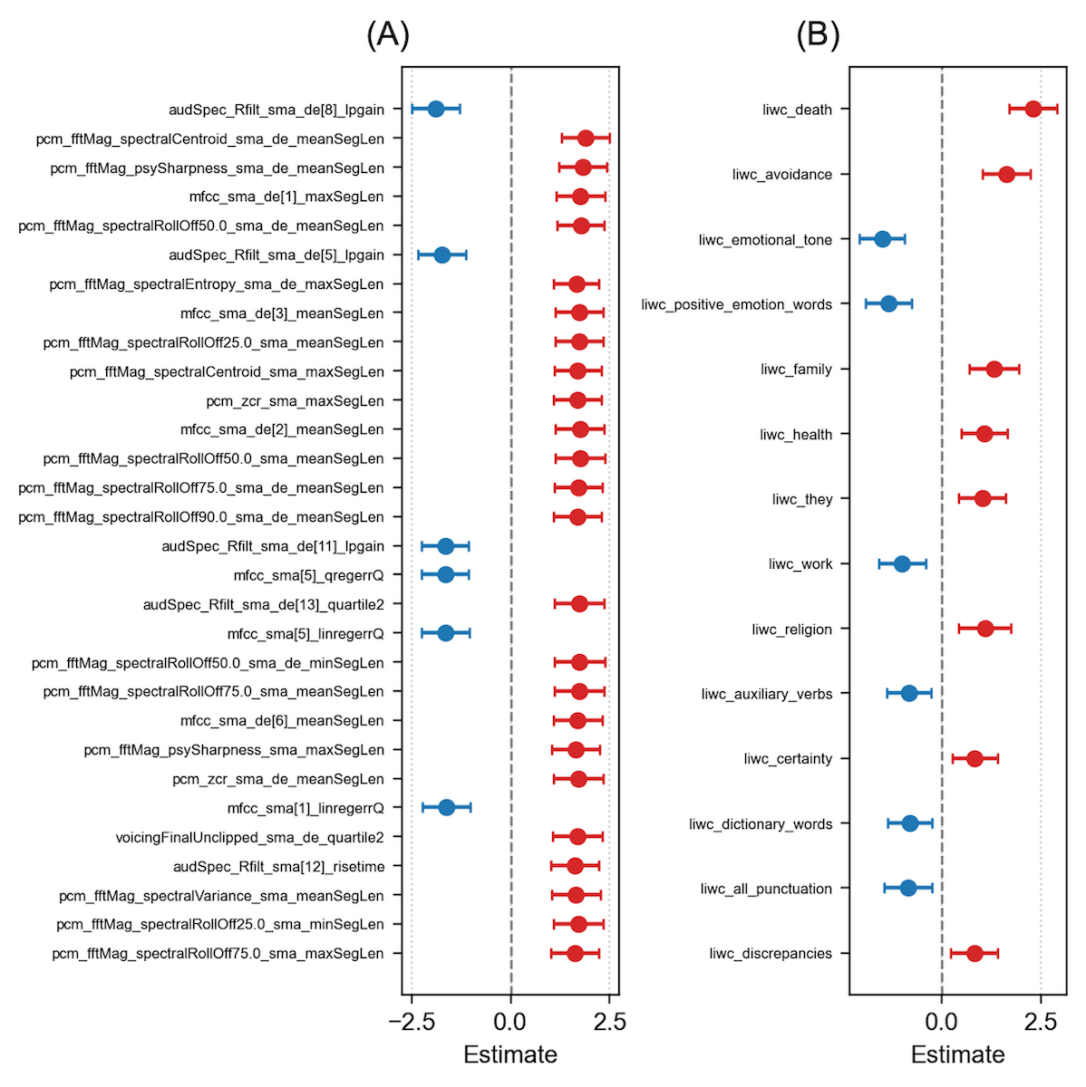
Forest plots displaying the results (β estimate) of mixed-effects multivariate linear regression between depression severity and (A) acoustic and (B) linguistic features. Only 30 acoustic features with the lowest *P* values are depicted for brevity. Red represents a positive association, whereas blue represents a negative association.

After adjusting for age, sex, BMI, and antipsychotic dosage and correcting for multiple testing, 1709 acoustic features were significantly associated with depression severity. The 5 most strongly associated acoustic features were audSpec_Rfilt_sma_de(8)_lpgain (linear prediction gain computed from the delta (first derivative) of the smoothed 8th RASTA-filtered auditory spectral band; β=−1.89; SE 0.31; Benjamini-Hochberg adjusted *P*<.001), pcm_fftMag_spectralCentroid_sma_de_meanSegLen (mean segment length of the time-derivative (delta) of the smoothed spectral centroid computed from the FFT-magnitude spectrum of the PCM signal; β=1.89, SE 0.31; adjusted *P*<.001), pcm_fftMag_psySharpness_sma_de_meanSegLen (mean segment length of the time-derivative (delta) of the smoothed psychoacoustic sharpness computed from the FFT-magnitude spectrum of the PCM signal; β=1.819, SE 0.31; adjusted *P*<.001), mfcc_sma_de(1)_maxSegLen (maximum segment length of the time-derivative (delta) of the smoothed first MFCC coefficient; β=1.765, SE 0.31; adjusted *P*<.001), and pcm_fftMag_spectralRollOff50.0_sma_de_meanSegLen (mean segment length of the time-derivative (delta) of the smoothed 50% spectral roll-off computed from the FFT-magnitude spectrum of the PCM signal; β=1.77, SE 0.30; adjusted *P*<.001; Multimedia Appendix 1). Participants with greater depression severity exhibited a reduced gain in relative spectral transform–filtered bands, which is related to speech clarity. The spectral centroid reflects the brightness of sound, whereas spectral roll-off measures the frequency below which 50% of the spectral energy is concentrated. Psychoacoustic sharpness captures the perceived clarity or harshness of the sound. Increased segment lengths in their temporal derivatives suggest prolonged periods of relatively unchanging frequency distribution, energy concentration, and perceptual sharpness (Figure 2A) [31].

After adjusting for age, sex, and years of education, 14 linguistic features were significantly associated with depression severity. The top 5 psycholinguistic categories associated with depression severity were “death” (β=2.307; SE 0.31; adjusted *P*<.001), “avoidance” (β=1.641; SE 0.30; adjusted *P*<.001), “emotional tone” (β=−1.50; SE 0.29; adjusted *P*<.001), “positive emotion” (β=−1.356; SE 0.30; adjusted *P*<.001), and “family” (β=1.32; SE 0.32; adjusted *P*<.001; Multimedia Appendix 2). Participants with depression more frequently used words related to death, avoidance, family, health, religion, negative emotions, and discrepancies and less frequently used words related to positive emotions and reward than their counterparts with minimal to mild depression (Figure 2B).

### Machine Learning Classification

#### Between-Person Classifier

**Figure 3:**
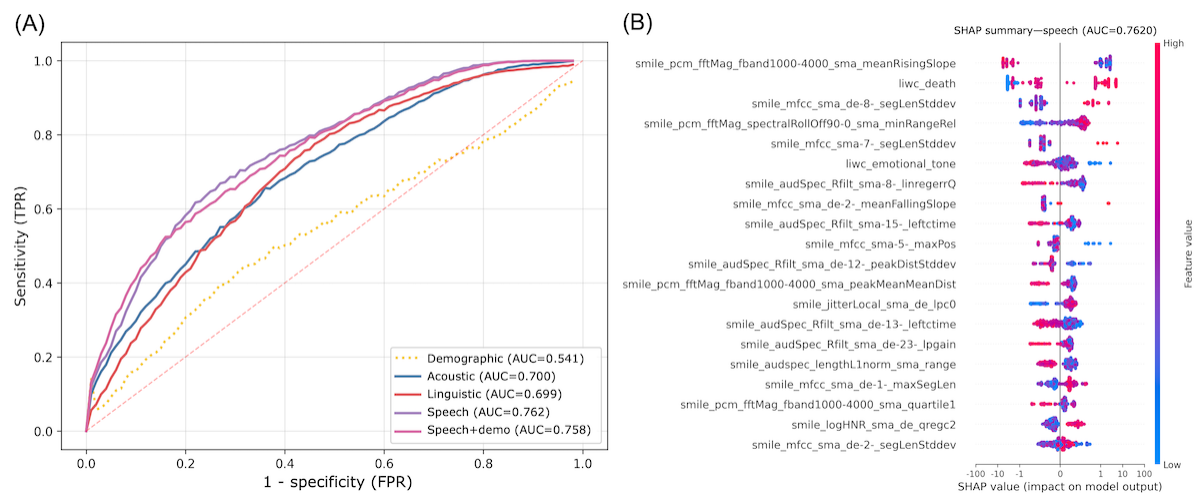
Between-person classification models for detecting moderate to severe depression: (A) receiver operating characteristic curves for light gradient boosting models (yellow line: demographic model [age and sex]; blue line: acoustic model; green line: linguistic model; purple line: speech model [acoustic+linguistic]; pink line: speech+demographic model) and (B) Shapley additive explanation (SHAP) summary plot for feature contributions for the best-performing model. AUC: area under the receiver operating characteristic curve; FPR: false positive rate; TPR: true positive rate.

Of 304 audio recordings, 129 (42.4%) were from patients with moderate to severe depression. Overall, LGB outperformed XGB. The best-performing classifier was the speech model (mean AUC 0.762, SD 0.002), followed by the speech+demographic model (mean AUC 0.758, SD 0.002). At the cutoff determined by the Youden index (0.30), the speech model achieved 72.5% sensitivity, 67.4% specificity, 69.6% accuracy, and a positive predictive value of 62%. Both acoustic and linguistic models outperformed the demographic model (AUC=0.700 and 0.699, respectively, vs AUC=0.541; Figure 3A).

SHAP analysis indicated that both acoustic and linguistic features contributed meaningfully to the inference of bipolar depression. Among linguistic features, a higher frequency of words related to death and lower emotional tone were associated with an increased likelihood of a positive (depressive) prediction. Acoustic predictors such as reduced variation in spectral slope and temporal instability increased the likelihood of a positive prediction (Figure 3B). To verify that the predictive importance of the LIWC “death” category was not driven by a small number of extreme cases, we examined its distribution (mean 0.00238, SD 0.00282). After excluding the recordings that exceeded the 3 SD cutoff (>0.01084; *z*=+3.04 to +5.55), the between-person model performance remained comparable or slightly improved (linguistic AUC=0.678 vs 0.699 before exclusion; acoustic AUC=0.781 vs 0.700; speech AUC=0.800 vs 0.762). These results confirm that the association between the “death” category and depression severity was not driven by outlier effects.

#### Within-Person Classifier for Predicting Recurrence

**Figure 4:**
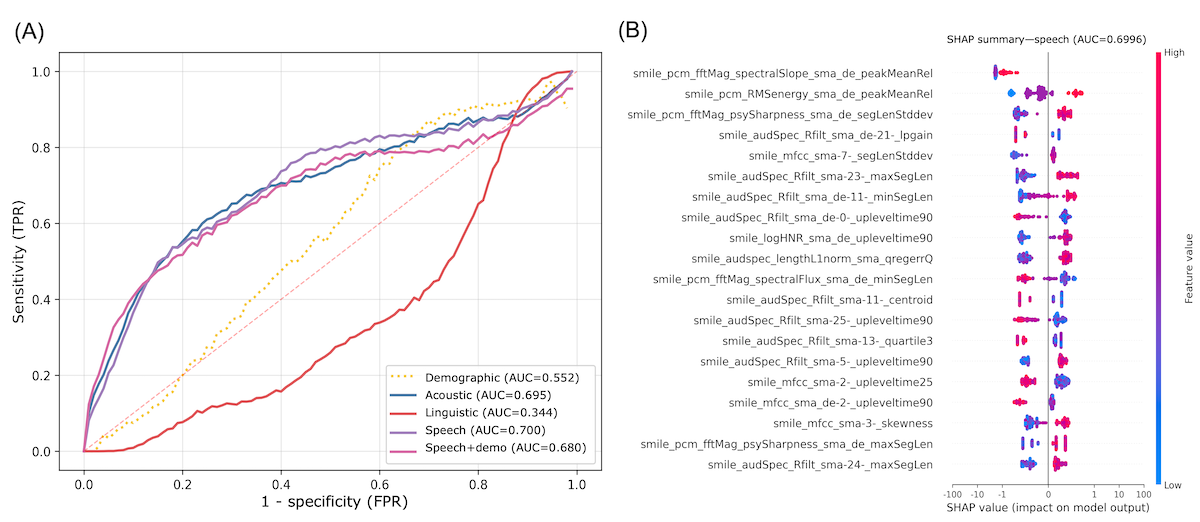
Within-person classification models for detecting recurrence: (A) receiver operating characteristic curves for extreme gradient boosting models (yellow line: demographic model [age and sex]; blue line: acoustic model; green line: linguistic model; purple line: speech model [acoustic+linguistic]; pink line: speech+demographic model) and (B) Shapley additive explanation (SHAP) summary plot for feature contributions for the best-performing model. AUC: area under the receiver operating characteristic curve; FPR: false positive rate; TPR: true positive rate.

The change in features from 212 paired audio recordings from consecutive visits was used, among which 24 (11.3%) follow-ups corresponded to recurrence events. XGB outperformed LGB. The best performance was achieved by the speech model, with a mean AUC of 0.700 (SD 0.003), accuracy of 80.6%, sensitivity of 49.3%, and specificity of 84.6% at the optimal cutoff of 0.10. The acoustic model achieved a comparable performance, with an AUC of 0.695 and accuracy of 0.835 (Figure 4). All performance metrics can be found in Multimedia Appendix 3.

SHAP analysis of the speech model suggests that the model relied primarily on acoustic features of reduced vocal energy, prosodic flattening, and articulatory slowing to infer depressive recurrence in bipolar disorder.

## Discussion

### Principal Findings

In this prospective longitudinal study of individuals with bipolar disorder, we demonstrated that both the acoustic and linguistic features of voice are strongly associated with depression severity and can be leveraged to accurately detect and monitor depressive states. In both between- and within-person analyses, models combining acoustic and linguistic speech features achieved the highest performance for identifying moderate to severe depression and predicting recurrence. In the between-person analysis, single-modality models using either acoustic or linguistic features outperformed the demographic-only (null) model, whereas in the within-person analysis, only the acoustic model showed superior performance over the null model. Incorporating demographic variables did not improve model performance in either task.

**Figure 5:**
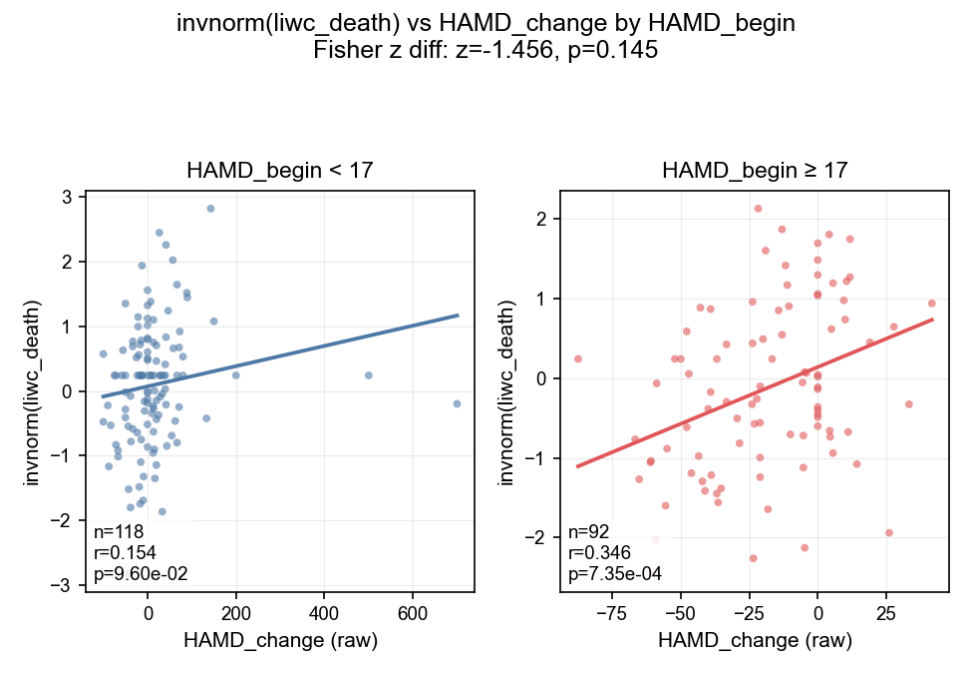
Correlation between changes in the Linguistic Inquiry and Word Count “death” category and changes in depression severity (Hamilton Depression Rating Scale) stratified by baseline severity. HAM-D: Hamilton Depression Rating Scale.

**Figure 6:**
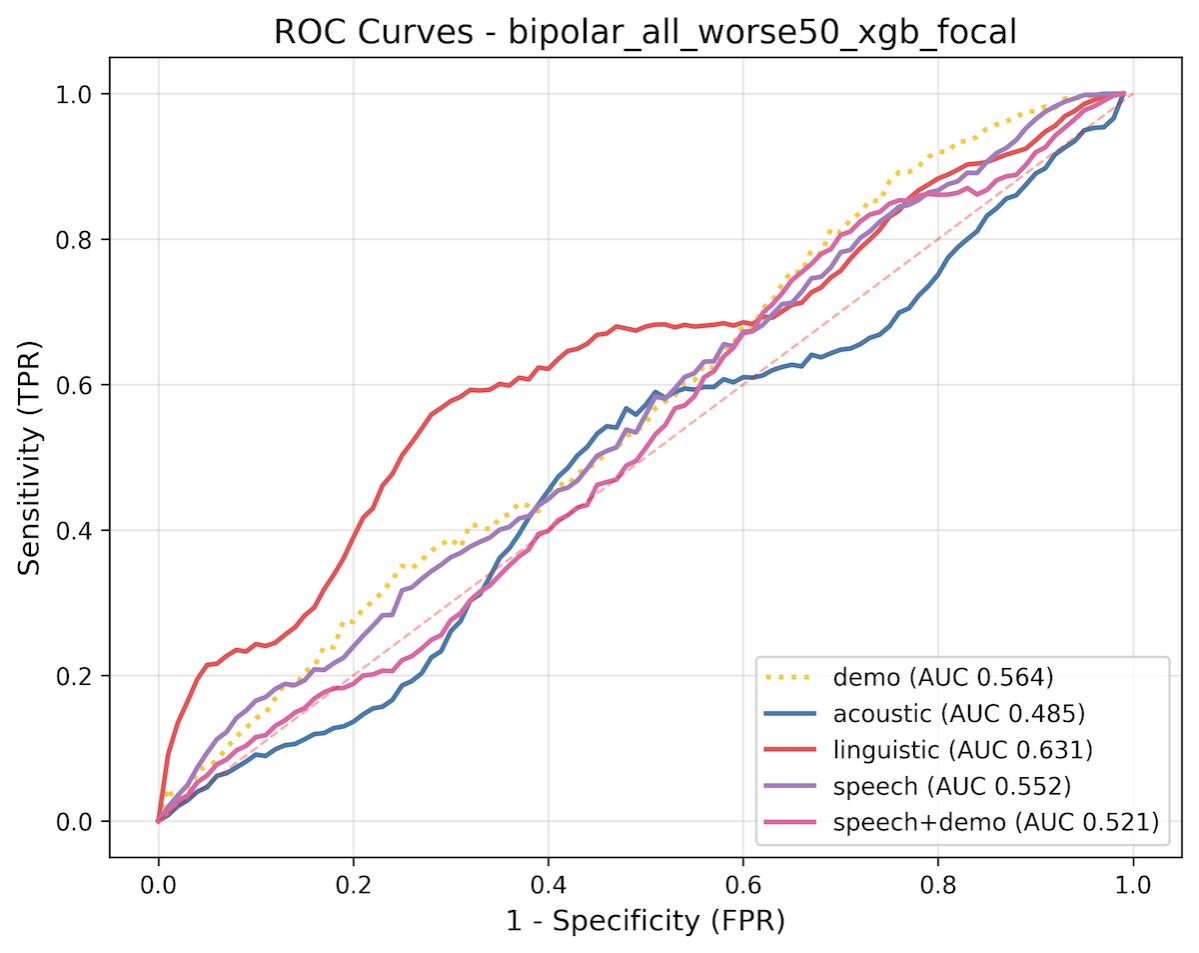
Receiver operating characteristic curves for the within-person classification models predicting recurrence defined as a ≥50% increase in Hamilton Depression Rating Scale score. The yellow line represents the demographic model (age and sex), the blue line represents the acoustic model, the green line represents the linguistic model, the purple line represents the speech model (acoustic+linguistic features), and the pink line represents the speech+demographic model. AUC: area under the receiver operating characteristic curve; FPR: false positive rate; ROC: receiver operating characteristic curve; TPR: true positive rate.

The within-person models did not outperform the between-person models. A major challenge was the substantial class imbalance, with recurrence occurring in only 11.3% (24/212) of follow-up visits. We addressed this by applying SMOTE to the training set within each cross-validation fold. Because participants who completed more visits contributed more paired samples to model training, these individuals may have exerted a greater influence on model learning. If attrition was nonrandom—if participants with sudden worsening were more likely to drop out—the within-person models may have been biased toward patterns characteristic of participants with better clinical course. We further examined the unexpected negative finding of the linguistic-only model. Changes in linguistic content were more pronounced among participants already in a severe depressive state where suicidal ideation and negative appraisals were more likely. For instance, the correlation between changes in death-related word use and HAM-D score change was stronger in those with a baseline HAM-D score of ≥17 than in those below the threshold (*r*=0.346 vs 0.154; Figure 5). Therefore, in many cases, the model would have predicted that greater increases in HAM-D scores were associated with worsening mood, yet these instances were labeled as negative because recurrence was defined as crossing the threshold from HAM-D<17 to ≥17. When recurrence was defined as a ≥50% increase in HAM-D score, thereby including the cases showing further worsening within the range of HAM-D≥17, the linguistic model’s performance improved (AUC=0.631; Figure 6), supporting our hypothesis.

Among the most important acoustic markers distinguishing depressed speech, we identified a reduced gain in the relative spectral transform–filtered spectral bands and longer continuous segments exhibiting changes in the spectral centroid, roll-off, and sharpness. These findings suggest that individuals with more severe depression display greater temporal monotony in the key spectral properties of their speech, which is consistent with the clinical observation of monotonous or less modulated vocal output in depression [5,8]. Numerous studies and meta-analyses have highlighted pitch-related features as one of the most robust acoustic markers of depression [5,6,8,15]. Changes in the fundamental frequency (F0), including reductions in mean pitch, pitch range, and pitch variability, have consistently been reported as characteristics of depressed speech. In our analysis, various pitch-related features were significantly associated with depression severity (Multimedia Appendix 1). Several metrics reflecting the temporal variability, range, and distributional characteristics of pitch change (ΔF0) were strongly associated with higher HAM-D scores.

Linguistically, individuals with depression used words related to death, avoidance, family, health, religion, negative emotions, and discrepancies more frequently, whereas positive emotions and reward-related words were less common. These linguistic features reflect the cognitive and affective themes of depressive states. Our findings closely align with meta-analytic results showing that depression is associated with increased use of negative emotion words and decreased use of positive emotion words [7]. Notably, although previous studies have highlighted increased first-person singular pronoun use as a linguistic marker of depression, no significant association between the use of first-person singular pronouns and depression severity was observed in our analysis. One explanation is that our study sample consisted of patients already diagnosed with bipolar disorder, which may have attenuated the discriminatory power of this marker. Additionally, the Korean language is known as one of the “pro-drop languages,” where pronouns are often omitted within sentences, especially when the subject, such as “I,” remains unchanged throughout the discourse [47]. Increased use of words related to death and health may reflect suicidal and somatic symptoms in patients with depression, whereas increased use of words related to avoidance and discrepancies and decreased use of reward-related words may reflect cognitive changes.

### Comparisons to Prior Work

While similar designs have demonstrated the potential for applying LIWC to automatically transcribed speech, this study implemented LIWC-based psycholinguistic analysis on automatically transcribed and machine-translated speech derived from psychiatric patient interviews. Previous research has applied LIWC to manually transcribed psychiatric interviews [36,48] or examined its use in artificial intelligence–generated transcripts in nonclinical populations [49]. However, none have used a fully automated pipeline combining both automatic speech recognition and machine translation in psychiatric populations. By advancing this methodology, our findings highlight the feasibility of scalable, cross-linguistic, multimodal speech-based digital biomarkers for depression.

Our study also included age, sex, antipsychotic dosage, and years of education as covariates in speech analysis. Previously, we have reported how antipsychotic medication can act as a confounder when analyzing changes in voice features associated with psychiatric symptoms, especially when drug-naïve individuals were used as controls [22,50]. Clinicians commonly prescribe or increase the dosage of antipsychotics when psychiatric symptoms worsen. Studies have reported that atypical antipsychotics affect vocal pitch and spectral features in a dose-dependent manner [50,51]. Years of education and speech content are also strongly associated with each other; individuals with fewer years of education produce less lexical and semantic content than those with higher levels of education [52]. Our covariate adjustments strengthened the robustness of our findings regarding the speech characteristics of patients with depression.

We implemented several strategies to mitigate overfitting, including BBCV with patient-level 5-fold cross-validation and 1000 iterations [43]. For linguistic analysis, we used the LIWC framework to quantify changes in the use of different psycholinguistic classes instead of counting the lexicons themselves. We considered the risk of overfitting by using a purely lexical approach, particularly when multiple recordings were collected from each participant. Previous studies have illustrated this limitation: Cohen et al [53] reported “yeah,” “very,” and “at” as top text model features, and Shin et al [54] reported “admission” as the single most important feature for detecting current depression, which is a finding unlikely to be replicated outside the tertiary hospital setting. To further assess the stability of our linguistic predictors, sensitivity analyses excluding recordings with extreme “death”-related word frequencies (>+3 SD) showed that between-person model performance remained comparable or slightly improved. This indicates that, rather than reflecting isolated outliers, the predictive contribution of death-related language represents a consistent and generalizable linguistic marker of depressive severity across participants.

### Limitations

This study has several limitations. First, all participants were diagnosed with bipolar disorder, which may limit the generalizability of our findings to other mood disorders. To assess the impact of diagnostic composition, we repeated all analyses after including patients with MDD. Including participants with MDD (44 recordings) did not substantially affect the between-person model performance (speech model AUC=0.751) but reduced the performance of the within-person model (speech model AUC=0.505; Multimedia Appendix 3). This finding suggests that consistent with the clinical distinction between bipolar and unipolar depression, their acoustic and linguistic patterns may diverge more prominently in the context of longitudinal recurrence prediction. The increased class imbalance introduced by including patients with MDD (from 7.83:1 to 8.38:1) may have also hindered classifier learning.

Second, because there is no established consensus on defining recurrence based on HAM-D scores, we adopted an operational definition of recurrence as a change from HAM-D<17 to 17 across 2 consecutive visits. This definition may have missed clinically meaningful fluctuations within the same severity range (eg, from 1 to 16 or from 17 to 24). We also tested an alternative definition of recurrence as a ≥50% increase in HAM-D scores; however, this approach did not improve classification performance (speech model AUC=0.552). Interestingly, the linguistic model was better trained at predicting 50% increase (AUC=0.631) than at predicting recurrence based on threshold crossing (AUC=0.344). This suggests that linguistic changes may be more sensitive to relative shifts in symptom severity than to categorical transitions in depression status.

Third, all interviews were conducted in a controlled environment, which may not reflect the variability in speech quality, background noise, or recording conditions encountered in real-world settings such as telemedicine or mobile apps.

Fourth, although participants were regularly followed up on in outpatient clinics and received appropriate care, we did not collect objective measures of hypomanic or manic symptoms, which may have influenced speech patterns.

Fifth, although antipsychotic dosage was included as a covariate in acoustic analyses, other psychotropic medications were not. Benzodiazepines can theoretically affect voice due to their muscle relaxation properties [55], and an early study reported that they can influence the modulation of the fundamental frequency of speech [56]. We found no empirical evidence that mood stabilizers affect voice acoustics. Although these medications were not included as covariates due to the limited evidence base, they may nonetheless represent potential confounding factors that warrant consideration in future work.

Sixth, the sample size was relatively small compared with the large number of features used in the classifiers. Nonetheless, a post hoc DeLong test incorporating the observed sample size, label distribution, and AUCs indicated statistical power exceeding 0.95 for feature comparisons against the demographic (null) model. We also applied BBCV to reduce overfitting. However, no independent external validation set was available at the time of our study.

Seventh, the sample was restricted to Korean-speaking individuals, which may constrain cross-cultural and cross-linguistic applicability. Given that acoustic and linguistic markers of depression may vary across languages and cultures, model performance may differ in more diverse populations.

Finally, we did not perform a manual validation of the translation accuracy. Therefore, potential translation bias may have influenced the distribution of specific LIWC categories, and this limitation should be considered when interpreting the linguistic results.

### Future Work

Replication in larger, independent samples—including different demographic groups, languages, and clinical settings—will be essential before widespread clinical implementation. While our study demonstrated the feasibility of applying LIWC-based analysis to machine–transcribed and translated speech, the impact of linguistic and cultural nuances on model accuracy can be further explored. Cross-cultural validation studies or fine-tuning of large language models on domain-specific corpora—such as annotated clinical interview transcripts—could enhance the accuracy and contextual relevance of linguistic feature extraction. Future studies should also compare speech-based biomarkers with other digital biomarkers and traditional clinical assessments to establish their relative utility and optimal integration strategies for comprehensive mental health monitoring platforms. Moreover, privacy and data security concerns are paramount when implementing such monitoring platforms in the real world based on biomarkers such as speech as voice can contain sensitive biometric information. With secure data pipelines, ethical governance frameworks, and clear clinical decision support protocols, speech-based mood monitoring can be effectively deployed across a spectrum of digital mental health tools, from virtual care services to AI-driven therapeutic platforms.

### Conclusions

Our findings provide evidence that both acoustic and linguistic features of speech serve as digital biomarkers of depression. By leveraging multimodal voice analysis and longitudinal modeling, we demonstrated the feasibility of scalable, automated, and noninvasive monitoring of depressive symptoms in real-world clinical practice. These results provide the groundwork for the future integration of speech-based tools into digital health platforms, offering new opportunities for personalized and continuous mental health care.

## Appendix

Multimedia Appendix 1 Associations between acoustic features and depression severity after adjusting for age, sex, BMI, and antipsychotic dosage.

Multimedia Appendix 2 Associations between linguistic features and depression severity after adjusting for age, sex, and years of education.

Multimedia Appendix 3 Performance metrics of all classification models.

## Abbreviations

AUC: area under the receiver operating characteristic curve
BBCV: bootstrap bias-corrected cross-validation
CER: character error rate
HAM-D: Hamilton Depression Rating Scale
LGB: light gradient boosting
LIWC: Linguistic Inquiry and Word Count
MDD: major depressive disorder
SHAP: Shapley additive explanation
SMOTE: synthetic minority oversampling technique
XGB: extreme gradient boosting

## Data Availability

The datasets generated or analyzed during this study are not publicly available due to the consent that did not cover public sharing of data, but the processed data are available from the corresponding author on reasonable request. Raw audio recordings could not be made available because of their sensitivity. Both the principal investigator (YMA) and first authors (SM and TY) had complete access to the data and took responsibility for their integrity.
